# Single candidate gene for salt tolerance of *Vigna nakashimae* (Ohwi) Ohwi & Ohashi identified by QTL mapping, whole genome sequencing and triplicated RNA-seq analyses

**DOI:** 10.1270/jsbbs.23053

**Published:** 2024-03-22

**Authors:** Miho Ito, Honami Ohashi, Masahiro Takemoto, Chiaki Muto, Takashi Seiko, Yusaku Noda, Eri Ogiso-Tanaka, Atsushi J. Nagano, Yu Takahashi, Jun Furukawa, Yuki Monden, Ken Naito

**Affiliations:** 1 Graduate School of Frontier Sciences, University of Tokyo, 5-1-5 Kashiwanoha, Kashiwa, Chiba 277-8561, Japan; 2 Research Center of Genetic Resources, National Agriculture and Food Research Organization, 2-1-2 Kannondai, Tsukuba, Ibaraki 305-8602, Japan; 3 Department of Agriculture, Okayama University, 3-1-1 Tsushimanaka, Okayama, Okayama 700-8530, Japan; 4 Takasaki Institute for Advanced Quantum Science, National Institutes for Quantum Science and Technology (QST), 1233 Watanuki-machi, Takasaki, Gunma 370-1292, Japan; 5 Center for Molecular Biodiversity Research, National Museum of Nature and Science, 4-1-1 Amakubo, Tsukuba, Ibaraki 305-0005, Japan; 6 Faculty of Agriculture, Ryukoku University, 1-5 Yokotani, Seta Oe-cho, Otsu, Shiga 520-2194, Japan; 7 Institute of Advanced Biosciences, Keio University, 403-1 Nipponkoku, Daihouji, Tsuruoka, Yamagata 997-0017, Japan; 8 Faculty of Life and Environmental Sciences, University of Tsukuba, 1-1-1 Tennodai, Tsukuba, Ibaraki 305-8572, Japan; 9 Graduate School of Environmental, Life, Natural Sciences and Technology, Okayama University, 3-1-1 Tsushimanaka, Okayama, Okayama 700-8530, Japan

**Keywords:** genetic resources, wild crop relatives, salt tolerance, QTL, whole genome sequencing, transcriptome analysis, genus *Vigna*

## Abstract

Salt tolerance has been an important issue as a solution for soil salinization and groundwater depletion. To challenge this issue, genetic diversity of wild plants must be harnessed. Here we report a discovery of a candidate gene for salt tolerance in *Vigna nakashimae*, one of the coastal species in the genus *Vigna*. Using intraspecific variation, we performed a forward genetic analysis and identified a strong QTL region harboring ~200 genes. To further narrow down the candidate genes, we performed a comparative transcriptome analysis, using the genome sequence of azuki bean (*V. angularis*) as a reference. However the detected differentially-expressed genes (DEGs) did not include those related to salt tolerance. As we suspected that the target gene in *V. nakashimae* is missing in *V. angularis*, we sequenced the whole genome sequence of *V. nakashimae* with long-reads. By re-analyzing the transcriptome data with the new reference genome, we successfully identified *POCO1* as a candidate gene, which was missing not only in *V. angularis* but also in the salt-sensitive accession of *V. nakashimae*. Further comparative analysis revealed that the tolerant genotypes conserved the ancestral form of the locus, while the sensitive genotypes did not. We also emphasize the pitfalls in our study, such as position effect in a growth chamber, missing important genes in the reference genome, and limited reproducibility of RNA-seq experiments.

## Introduction

Developing salt-tolerant crops is one of the important issues in agriculture. One problem is saline or sodic soil, which has increased by ~270 Mha in the last 30 years (Hassani *et al.* 2020). The other problem is a shortage of fresh water, which is mainly caused by depleting groundwater for irrigation (Wada *et al.* 2010). In addition, terrestrial water storage is also declining due to climate change (Pokhrel *et al.* 2021, Yao *et al.* 2023). Thus, there has been a demand for salt-tolerant crops that can be cultivated in salt-affected land or with salinized water (Panta *et al.* 2014).

To tackle these challenges, it is necessary to harness the genetic diversity of wild genetic resources (McCouch *et al.* 2013). Although there have already been lots of studies on salt tolerance, having identified more than 600 salt-responsive genes, little is known regarding how to develop a salt-tolerant crop with those genes. This is because most of such studies are of model plants, which are sensitive to salt stress. Thus, it is important to elucidate the mechanisms of wild plants that are adapted to saline environments such as marine beaches.

The salt-tolerant species of the genus *Vigna*, which we have identified previously (Iseki *et al.* 2016, Yoshida *et al.* 2016), could represent such wild plants. The important features of these species are not only the high tolerance to salt stress, but also the diverse and unique mechanisms of salt tolerance they have evolved (Noda *et al.* 2022). In addition, Vigna species are mostly diploid and have relatively small genomes (~500 Mbp), making genomic studies affordable.

Here we present genetic, genomic and transcriptomic analyses on one of the salt-tolerant species, *Vigna nakashimae* (Ohwi) Ohwi & Ohashi. This species exhibited the best performance in some criteria in our previous studies (Iseki *et al.* 2016, Yoshida *et al.* 2016), suppressing sodium allocation to the leaf and maintaining low Na/K ratio (Noda *et al.* 2022). Interestingly, although almost all the accessions of *V. nakashimae* are more tolerant than other relatives of azuki bean, there is still variation in salt tolerance within the species. In a study by Yoshida *et al.* (2016), some Korean accessions exhibited salt injuries within 1 week under 200 mM NaCl, while the accession collected from Ukushima Island in Japan did not for at least 2 weeks. Thus, we crossed the tolerant accession with the relatively sensitive accession and performed a classic, forward-genetic analysis. Using the azuki bean genome v1.0 (Sakai *et al.* 2015a) as a reference, we obtained genetic markers and detected a strong QTL peak for salt tolerance. However, the following transcriptomic analysis failed in identifying candidate genes. As we suspected that our target gene was missing in the azuki bean genome, we sequenced and assembled the whole genome of *V. nakashimae*, reanalyzed the transcriptome data and finally identified a single candidate gene. In addition to the possible role of the candidate gene in salt tolerance, we will discuss some pitfalls we have encountered during the study, including position effect of plant growth in a growth chamber, lack of target genes in the genomes of related species, and limited reproducibility of transcriptome results.

## Materials and Methods

### Plant materials

Plants studied here, all provided by NARO genebank (https://www.gene.affrc.go.jp/index_en.php) are listed in Table 1. To develop a mapping population, we cultivated F_1_ plants derived from JP254478 × JP247291 and harvested F_2_ seeds. We also cultivated 200 F_2_ plants, collected young leaves from each plant for DNA extraction, and harvested F_3_ seeds. Plants in all other experiments were grown in hydroponic culture which is described below.

### Tracer experiment of ^22^Na

The seeds of JP254478 and JP247291 were sterilized by shaking in 70% ethanol for 5 min and in 5% sodium hypochlorite for 5 min. The sterilized seeds were scratched, embedded in a bed of Seramis Clay Granules (Effem GmbH, Verden, Germany) and incubated in a growth chamber with temperature of 28°C for 1 week. The germinated seedlings were transplanted to hydroponic culture containing 1× Otsuka house No. 1 and 1× Otsuka house No. 2 (Otsuka Chemical Co., Osaka, Japan). Plants were grown in a walk-in growth chamber (CWH-40A, ESPEC MIC Corp.) with 14 h light/10 h dark cycle at 28°C. In a treatment of salt stress, plants were transferred to hydroponic culture with 50 mM NaCl for 3 days. After pretreatment with 50 mM NaCl, JP254478 and JP247291 were transferred to a new hydroponic culture with 5 kBq ^22^Na (PerkinElmer, USA) with non-radioactive 100 mM ^23^NaCl, and were incubated for another 3 days in a growth chamber as described above. When sampling, the root of the plants was carefully washed. The collected plants were enclosed into a plastic bag and exposed to a Storage Phosphor Screen (BAS-IP-MS-2025E, GE Healthcare, UK) in Amersham exposure cassettes (GE Healthcare, UK) for 24 h. By scanning the phosphor screen with a laser imaging scanner Typhoon FLA-9500 (GE Healthcare, UK), images indicating intensity of radioactivity were obtained. All the experiments were independently triplicated with more than three biological replicates.

### Genotyping and linkage map construction

We extracted DNA of the parental accessions and F_2_ plants from the collected leaves by a CTAB method. We then screened the 316 SSR markers of azuki bean (Kaga *et al.* 2008) for polymorphism between the parental accessions (Supplemental Table 1). The selected markers were used to genotype 190 F_2_ plants by fragment analysis as described by Isemura *et al.* (2012).

We also performed restriction-associated DNA tag sequencing (RAD-seq) to further genotype the F_2_ plants as described by Sakaguchi *et al.* (2015). Briefly, we digested 10 ng DNA with *Eco*RI and *Bgl*II (New England Biolabs, Ipswich, MA, USA), and ligated the adapters to the digested DNA. The adapter-ligated DNA was purified with AMPure XP (Beckman Coulter, CA, USA), indexed and amplified by PCR with KOD-Plus-Neo (TOYOBO, Osaka, Japan). The PCR products were pooled, purified with AMPure XP and sequenced with 2 lanes of 51 bp single-end module of Illumina HiSeq2000 (Illumina, San Diego, CA, USA) by Macrogen (Seoul, South Korea). The obtained sequences were mapped to azuki bean genome v1.0 (Sakai *et al.* 2015a) by bwa-mem2 (Vasimuddin *et al.* 2019) with default settings, and processed by ref_map.pl pipeline of STACKS-2.41 (Rochette *et al.* 2019) with an option of -X “populations: --map-type F_2_ --map-format onemap” to obtain ABH genotype table. From the genotype table, we removed loci with more than 10% missing or with distorted segregation (p < 1^–10^ in the chi-square test).

To construct a genetic linkage map, we used onemap package (https://doi.org/10.1101/2022.11.24.517847) in R software. We followed the whole process described in the tutorials (https://statgen-esalq.github.io/tutorials/onemap/Inbred_Based_Populations.html). We note that, to run one map, the genotypes in the raw file must be in capital letters, though those generated by STACKS are in lower case letters.

### Phenotyping and QTL analysis

To evaluate the salt tolerance of each F_3_ line in the mapping population, we cultivated 6 plants of each line and treated them with salt stress as follows. The seeds were sterilized and germinated as described above. The germinated seedlings were transplanted in six hydroponic culture pools containing 1× Otsuka house No. 1 and 1× Otsuka house No. 2 (Otsuka Chemical Co., Osaka, Japan), with each pool harboring 1 plant per line. Plants were pre-treated with 50 mM NaCl as described above. The plants were then transferred to new hydroponic culture with 200 mM NaCl. After 10 days of salt stress treatment, we visually evaluated the damage scores of the primary (first) leaves with “0” as no damage, “1” as less than 50% damaged, “2” as more than 50% damaged, and “3” as dead or detached. The damage score of each plant was the average of those independently evaluated by 4 persons. The damage score of each F_3_ line was given by further averaging the scores of the 6 plants. The averaged damage scores of F_3_ lines are taken as the phenotypes of the F_2_ plants for QTL analysis.

In addition, we performed the phenotyping experiment twice. In the first experiment, we evaluated 108 F_3_ lines but carelessly placed the plants of each line in the same positions in the pools. The difference in the salt damage between the plants placed at the edge of the pools and those placed inside were tested by Mann-Whitney U test.

In the second experiment, we evaluated the salt damage of another 82 F_3_ lines with randomized positions to minimize position effect within a pool.

Together with the genotypes and the linkage map, we performed QTL analysis using qtl2 package (Broman *et al.* 2019) of R software, according to the user guide (https://kbroman.org/qtl2/assets/vignettes/user_guide.html). Although additive effect (a) and dominance effect (d) can be estimated with qlt2, the function to estimate proportion of phenotypic variance explained (PVE) is deprecated. Thus, we estimated PVE as follows. 1) Predicted phenotype of each F_3_ line was estimated based on the genotype (number of sensitive allele), additive effect (a) and dominance effect (d). If the genotype is sensitive homo or tolerant homo, the predicted phenotype was given as 2*a or 0*a, respectively. If heterozygous, it was given as 1*a + d. Genetic variance (Vg) of F_3_ lines was then calculated from the predicted phenotypes. PVE was given as Vg/Vp, where Vp is phenotypic variance which was given as the variance of the observed damage scores in the F_3_ lines.

### RNA-seq analysis

We performed RNA-seq analysis 3 times. In the first experiment, the plants of JP254478 and JP247291 were cultivated as described above, pre-treated with 50 mM NaCl for 3 days and then treated with 200 mM NaCl for 3 days. In the second experiment, the plants of all the five accessions in Table 1 were treated with 100 mM NaCl for 3 days, without pre-treatment. The third experiment was done as in the first one. Each experiment also had control plants cultivated without salt stress.

After stress treatment, we collected leaf samples and root samples from each plant. The collected samples were immediately frozen in liquid nitrogen and stored in –80°C until RNA extraction, which was done with RNeasy Plant Mini Kit (Qiagen K.K, Tokyo, Japan). In the first experiment, non-stranded mRNA-seq was done with HiSeq2000. Library prep and sequencing were both provided as a custom service by Macrogen. In the second and the third experiments, we prepared the library with Collibri 3′ mRNA-seq Library Prep Kit for Illumina Systems (Thermo Fisher Scientific, MA, USA) and had the library sequenced with HiSeq4000 by GeneBay Inc. (Yokohama, Japan).

We used Salmon (Patro *et al.* 2017) to obtain raw counts of each gene from RNA-seq data according to the standard protocol. The obtained read counts were then processed with edgeR (McCarthy *et al.* 2012) to identify differentially expressed genes (DEGs) as described by Chen *et al.* (2016). We compared the transcription of the leaf or the root of JP247291 vs JP254478 under salt stress. DEGs had to satisfy all the four criteria as follows; [log2FC > 1 or <–1], [mean CPM > 1], [p < 0.05], and [FDR < 0.05], where logFC, mean CPM, p and FDR indicate log2 transformed fold change, average CPM of all samples, p value and false discovery rate, respectively.

All the three experiments were done with three biological replicates.

### Whole genome sequencing

We sequenced and assembled the whole genomes of JP254478 and JP247291 as described by Naito (2023). Briefly, we extracted DNA from unexpanded leaves with Nucleobond HMW DNA Kit (Takara Bio, Kusatsu, Japan), prepared library with Ligation Sequencing Kit SQK-LSK112 (Oxford Nanopore Technologies, Oxford, UK), and sequenced with an R10.4 (Oxford Nanopore Technologies, Oxford, UK) flow cell in PromethION 24 (Oxford Nanopore Technologies, Oxford, UK). We also obtained 150 bp paired-end reads with HiSeq4000 by Gene Bay Inc. We assembled the long-reads with NECAT (Chen *et al.* 2021), polished once with medaka-1.8.1 (https://github.com/nanoporetech/medaka) using long-reads only and twice with HyPo (https://doi.org/10.1101/2019.12.19.882506) using short-read only. We then discarded heterozygous or repetitive contigs by purge_haplotigs (Roach *et al.* 2018). The assembled contigs were anchored to the linkage map to reconstruct pseudochromosomes.

We also annotated the genomes with presumed ORFs by Braker-2.3 (Brůna *et al.* 2021) and GeMoMa-1.9 (Keilwagen *et al.* 2018) according to the standard protocols. We removed overlapped annotations across the annotation tools according to output of GffCompare (Pertea and Pertea 2020). Of the remaining annotations, we retained those categorized as “Viridiplantae” or “Eukaryotes” by EnTAP (Hart *et al.* 2020).

### Data availability

All the sequence data are available with Biosample IDs SAMN36705140-SAMN36705403, under Bioproject PRJNA998483. The assembled genome sequence and annotations are available from Vigna Genome Server (https://viggs.dna.affrc.go.jp/download_Vnakashimae_v1) (Sakai *et al.* 2015b).

## Results

### Sodium allocation in the parental accessions

**Fig. 1.**
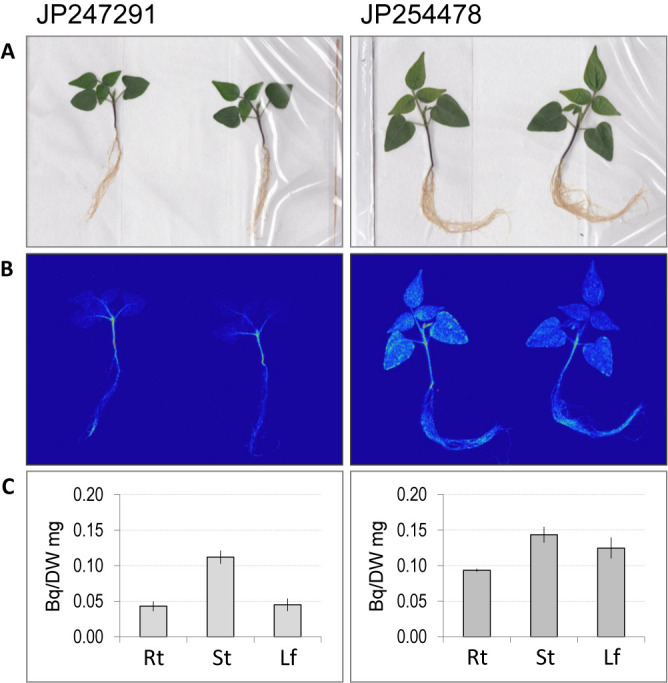
Na allocation in the plants of parental accessions. A. Photograph of JP247291 and JP254478 fed with ^22^Na. B. Autoradiograph of JP247291 and JP254478 fed with ^22^Na. C. Amounts of ^22^Na per biomass. Y-axis indicates radioactivity (Bq) per gram dry weight. Rt, St and Lf indicate roots, stems and leaves, respectively. Error bars indicate standard deviation.

To reveal if there is difference in sodium allocation between the tolerant accession (JP247291) and the sensitive accession (JP254478), we obtained autoradiography of the both accessions by feeding the plants with radioactive Na (^22^Na). As a result, JP247291 reproduced the pattern of sodium allocation in our previous study (Noda *et al.* 2022), where ^22^Na was mainly allocated in the stem and was not allocated to the leaf (Fig. 1). In contrast, ^22^Na was accumulated in the whole plant body in JP254478, with ~2.5 times higher concentration in the leaf than that of JP247291 (Fig. 1).

### Difference of salt tolerance between the parents

Before evaluating the mapping population, we performed a preliminary experiment with the parental accessions to decide the intensity and duration time of salt stress (Supplemental Fig. 1).

Without salt stress, there was no difference in the progress of the development stage between the 2 accessions, but JP254478 always grew bigger than JP247291. In 50 mM or 100 mM NaCl, we observed no damage in either of the accessions at least for 10 days.

In 150 mM NaCl, JP247291 still displayed no salt damage for 10 days. In JP254478, the primary leaves (the first leaf that are usually unifoliate) began to display damage in 6 days and those in some plants were wilted or detached in 10 days. However, the true leaves (the 2nd or later leaves that are usually trifoliate) displayed no damage for 10 days.

In 200 mM NaCl, the primary leaves of JP247291 plants began to display salt damage in 5 days but none were wilted or detached in 10 days. In contrast, those in JP254478 were damaged in 3 days and were all wilted or detached within 10 days. As for true leaves, JP247291 displayed no damage while some of JP254478 plants were damaged or wilted. However, the damage in true leaves highly varied across individuals, making it difficult to stably evaluate the salt damage. Thus, we decided to use salt damage of the primary leaves in 200 mM NaCl for 10 days to evaluate damage scores of the mapping population.

### Linkage map

We screened for polymorphic markers from the 316 SSR markers that were once used for constructing a linkage map of azuki bean (Kaga *et al.* 2008). Of the 316, 101 were polymorphic but we selected 54 of them as the remaining 47 showed overlapping genotypes with others (Supplemental Table 1). With the selected markers, we successfully genotyped 190 F_2_ plants.

In addition, we performed RAD-seq to obtain more markers to cover the whole genomic regions. The sequence data revealed 1,485 polymorphic SNP loci but 1,274 were filtered because of too many missing genotypes, too much distorted segregation, or completely overlapped genotypes with other SNP loci.

In total, we obtained 264 marker loci and performed linkage analysis (Supplemental Tables 2, 3). Although the karyotype of *V. nakashimae* is 2n = 22, we obtained only ten linkage groups (Fig. 1B). Most of the linkage groups corresponded to the respective chromosomes (Supplemental Fig. 2), but the largest linkage group, designated as “1 + 7”, consisted of markers derived from chromosomes 1 and 7 of azuki bean (Supplemental Fig. 2). As observed in linkage maps by Kaga *et al.* (2008) and Wang *et al.* (2015), this could be a sign of reciprocal translocation between these chromosomes.

### QTL analysis

**Fig. 2.**
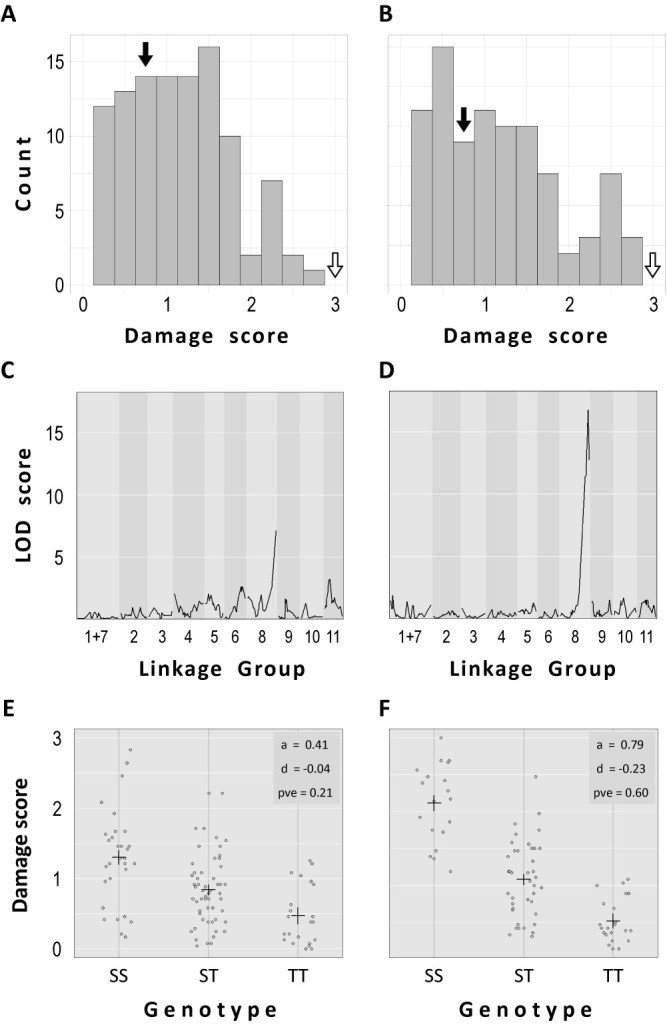
Summary of QTL analysis of the first experiment (A, C, E) and the second one (B, D, F). A, B. Histogram of damage score in the mapping population. The black and white arrows indicate the damage score of JP247291 and JP254478, respectively. C, D. LOD curves estimated by Haley-Knott regression. E, F. Distribution of damage score by genotype at QTL peak. S and T indicate alleles derived from the sensitive and tolerant parent (JP254478 and JP247291), respectively.

We cultivated 108 F_3_ lines, 6 plants per line, in 200 mM NaCl for 10 days (Supplemental Table 4). The distribution of damage scores (average of the 6 plants) was as shown in Fig. 2A. With the damage scores as phenotype values, we performed QTL analysis and detected a single peak with a LOD score of 6.7 at the marker “M193”, which was located at 105.02 cM on chromosome 8 (Fig. 2C, Supplemental Table 2). The additive effect of the QTL was 0.41 and the dominance effect was –0.04, explaining 21% of phenotypic variance (Fig. 2E).

However, we noticed that the plants placed at the edge of a culture pool tended to display more damage compared to those placed inside (Supplemental Fig. 3). Unfortunately, we were not careful enough to randomize the position of each line. Since the Mann-Whitney U test revealed that the damage scores between the lines positioned at the edge and those in the middle were significantly different (Supplemental Fig. 4), we even suspected that the detected QTL peak was a false positive.

To test if the position effect disturbed the QTL analysis above, we performed another phenotyping experiment with 82 F_3_ lines (Supplemental Table 4). The distribution of damage scores was more bipolarized with higher frequencies of higher or lower damage scores (Fig. 2B). The following QTL analysis detected, even with fewer lines tested, a much higher peak (LOD score of 17.1) at the same position as in the first analysis (Fig. 2D). Correlation between the damage scores and the genotypes in the QTL was much stronger than that in the first experiment, with additive and dominance effects of 0.79 and –0.23, respectively (Fig. 2F). In addition, this QTL explained 60% of phenotypic variance (Fig. 2F).

In both QTL analysis, the QTL peak ranged from 96.8–110.02 cM region on chromosome 8, which corresponded to a genomic region of 44,326,269–46,835,504 nt, harboring 214 genes (Supplemental Table 5).

### Transcriptome analysis

To narrow down the candidate genes of salt tolerance, we performed transcriptome analysis on the salt-treated plants of the parental accessions. To minimize falsely-detected DEGs, we performed RNA-seq three times. Here, the genes with significantly higher or lower expression in JP247291 than in JP254478 were defined as up-regulated genes or down-regulated genes, respectively.

**Fig. 3.**
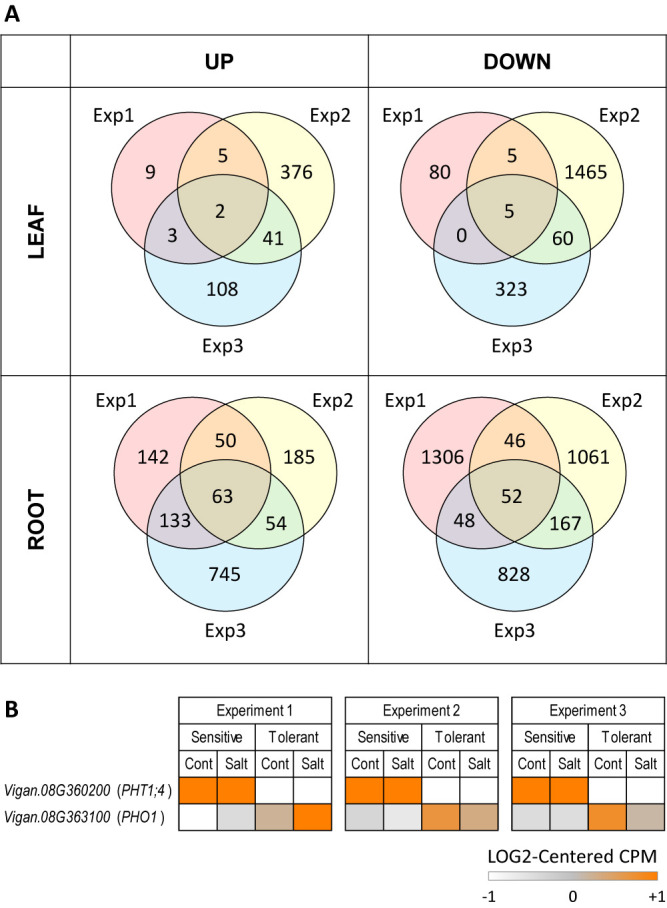
Summary of transcriptome analysis based on reference genes of azuki bean. A. Venn diagrams of significantly up- or down-regulated genes detected in the leaf or in the root by the 1st, 2nd and 3rd experiments. B. Heatmap of DEGs located within the QTL region. In each experiment, “Sensitive” and “Tolerant” indicate JP212346 and JP247291, respectively. “Cont” and “Salt” indicate the control and salt-stressed condition, respectively.

Although there were hundreds of up-regulated and down-regulated genes in each experiment, few were overlapped across the three experiments (Fig. 3A, Supplemental Tables 5–7). In the leaf, there were only 7 DEGs (2 up-regulated and 5 down-regulated) that were significant throughout the three experiments and none of them were located within the QTL region. In the root, there were 115 DEGs (63 up-regulated and 52 down-regulated) that were significant throughout the three experiments. Of them, one up-regulated and one down-regulated gene were located within the QTL region (Fig. 3B, Supplemental Tables 6, 7).

The up-regulated gene within the QTL was *Vigan.08G363100.01*, encoding PHOSPHATE 1 (PHO1), and the down-regulated gene was *Vigan.08G360200.01*, encoding PHOSPHATE TRANSPORTER 1;4 (PHT1;4). Given PHO1 and PHT1;4 are both related to phosphate transport, it seemed unlikely that these genes were related to salt tolerance (Fig. 3B, Supplemental Table 5).

As such, we considered other approaches were necessary to identify the candidate gene for the salt tolerance in JP247291 of *V. nakashimae*.

### Genome sequence of *V. nakashimae*

Since the results of transcriptome analysis seemed to have failed in narrowing down the candidate genes, we suspected that the QTL region in *V. nakashimae* genome contains genes that are missing in the corresponding region of azuki bean genome, which was used as a reference for the transcriptome analysis. To overcome such problems, we *de novo* assembled the whole genome sequences of both JP247291 and JP254478.

**Fig. 4.**
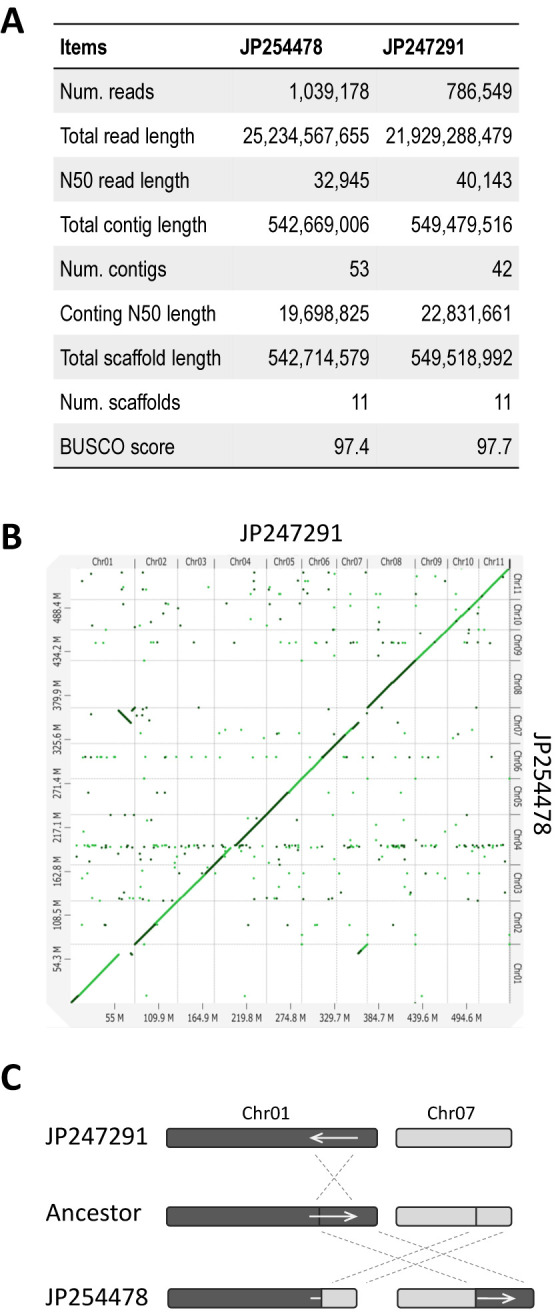
Summary of *V. nakashimae* genome. A. Statistics of sequencing and assemblies of JP254478 and JP247291. B. Dotplot of pseudochromosomes between JP242791 and JP254478. C. Schematics of reciprocal translocation and inversion in chromosome 1 and chromosome 7. Arrows indicate inverted regions in JP242791.

With 30–50x coverage of nanopore long-reads, the draft assemblies of JP247291 and JP254478 were assembled into 42 and 53 contigs, respectively (Fig. 4A). More than 99% of the draft assemblies were anchored to pseudochromosomes, leaving only 4 contigs (1.6 Mbp) in JP247291 and 20 contigs (5.3 Mbp) in JP254478 unplaced (Fig. 4A). We also achieved high-quality gene annotation, as the protein BUSCO scores reached 97.7 for JP247291 and 97.4 for JP254478 (Fig. 4A).

The assembled genome sequence revealed a reciprocal translocation between chromosomes 1 and 7, which was also suggested during the linkage analysis (Figs. 1B, 4B). The translocation event should have occurred in JP254478, as the chromosomes 1 and 7 of JP247291 conserved collinearity with those of azuki bean. We also found an inversion that was specific to chromosome 1 of JP247291 (Fig. 4B, 4C).

In the new reference genome of JP247291, the corresponding region of the QTL was 58,074,882–59,777,341 nt on chromosome 8, harboring 159 genes (Supplemental Table 8).

### Transcriptome analysis with new reference

**Fig. 5.**
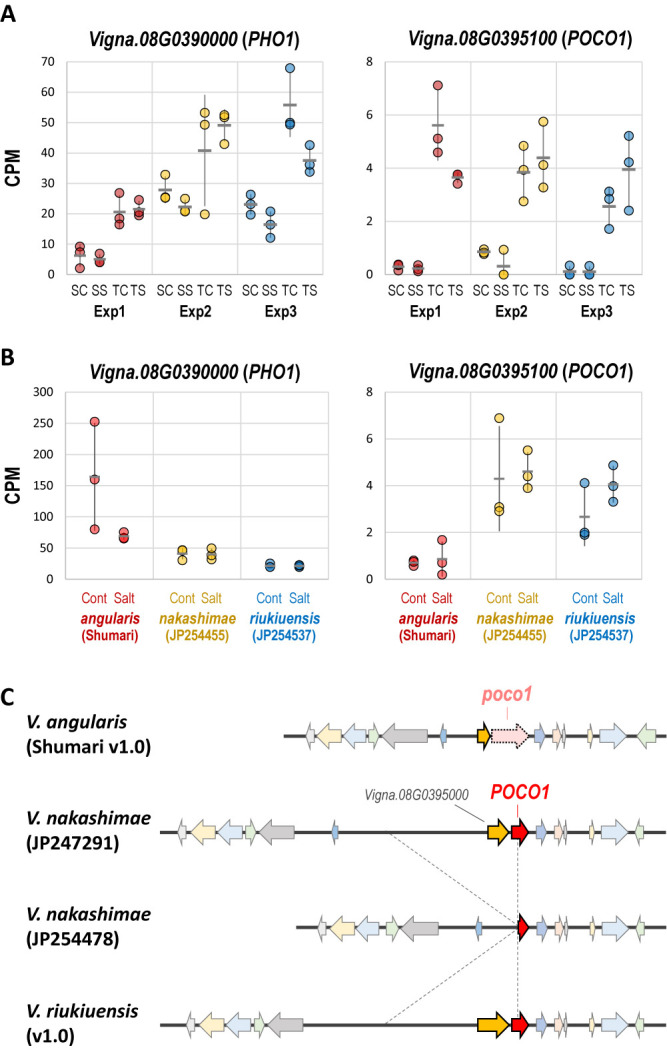
Transcript abundance of the candidate genes and genomic structures around *POCO1* locus across species. A. Transcript abundance of *PHO1* and *POCO1* genes in the root of JP254478 (sensitive) and JP247291 (tolerant). Exp1, Exp2 and Exp3 indicate the 1^st^, 2^nd^ and 3^rd^ experiments. SC, SS, TC, TS indicate sensitive in control, sensitive in salt, tolerant in control and tolerant in salt, respectively. Circles, horizontal lines and vertical lines indicate CPM values of biological replicates, means and standard deviations, respectively. B. Transcript abundance of *PHO1* and *POCO1* genes in the root of cv. ‘Shumari’, JP254455 and JP254537. Cont and Salt indicate the control and salt-stressed conditions, respectively. Circles, horizontal line and vertical lines indicate CPM values of biological replicates, means and standard deviations, respectively. C. Schematic of genomic structures around *POCO1* locus. Arrows indicate ORFs and orthologous genes are indicated with the same colors. Dotted lines indicate the deleted region in JP254478.

Using the genome sequence of JP247291 as a new reference, we re-analyzed the RNA-seq data (Supplemental Tables 8–10). The overall result was similar to that with azuki bean genome and, within the QTL region, there were no DEGs in the leaf that were significant across the three experiments. However, from the root, we detected two DEGs within the QTL that were both up-regulated across the three experiments (Fig. 5A, Supplemental Tables 8–10). One was *Vigna.08G0390000.01*, which encoded PHO1 and was also detected in the first analysis, but the other was *Vigna.08G.0395100.01*, which encoded PRECOCIOUS1 (POCO1) and was detected only in this analysis.

In both *PHO1* and *POCO1*, the abundance of transcripts was higher in JP247291 than in JP254478, both in the control and the salt-stressed conditions (Fig. 5A). Although the *PHO1* was more transcribed (CPM values of 5–70), the difference was more dramatic in *POCO1* as the CPM values were less than 1 in JP254478 (Fig. 5A, Supplemental Table 8).

To further elucidate which of the two genes was more likely to be the candidate, we also performed RNA-seq on *V. angularis* (cv. ‘Shumari’, salt-sensitive), *V. riukiuensis* (JP254357, salt-tolerant) and another accession of *V. nakashimae* (JP254455, salt-tolerant). As a result, the abundance of transcripts in *PHO1* was the highest in *V. angularis*, the most salt-sensitive accession among the five tested in this study (Table 1, Fig. 5B). In contrast, the abundance of transcripts in *POCO1* exhibited a positive correlation with salt tolerance (Table 1, Fig. 5B). The CPM values were less than 1 in *V. angularis* and were 3–6 in JP254455 and JP254537.

We also wondered why *PHT1;4* was not detected as a DEG within the QTL in this re-analysis. By looking through the *V. nakashimae* genome, we found there were 4 loci encoding *PHT1;4* (*Vigna.04G0340900.01*, *Vigna.04G0341300.01*, *Vigna.04G0341500.01*, and *Vigna.08G0125400.01*), none of which were located within the QTL region, indicating *PHT1:4* was a false positive in the previous analysis where we used azuki bean genome as a reference.

### Structural variation in *POCO1* locus

As we also assembled the whole genome of JP254478, we compared the sequences around the *POCO1* (*Vigna.08G0395100.01*) locus of the two accessions, together with those of azuki bean genome v1.0 (Sakai *et al.* 2015a) and *V. riukiuensis* genome v1.0 (https://doi.org/10.1101/2022.03.28.486085).

The results revealed that JP254478 harbored a large deletion (Fig. 5C). The deleted sequence included the whole locus of *Vigna.08G0395000.01*, which is a neighbor of *POCO1*, and the 1st half of *POCO1* locus. In addition, the *POCO1* locus of *V. angularis* accumulated repetitive sequences, presumably making it a pseudogene (Fig. 5C). *V. riukiuensis* conserved exactly the same structure as JP247291 (Fig. 5C), indicating these two accessions retained the ancestral form of this region, whereas *V. angularis* and JP254478 had the derived forms.

We also suspected that *Vigna.08G0395000.01*, which was completely deleted in JP254478 (Fig. 5C), could be another candidate gene, but we ruled out the hypothesis as no transcripts of this gene were detected at all from any of our RNA-seq data.

### Other differentially expressed genes

Given *POCO1* is involved in ABA signaling (Emami and Kempken 2019, Emami *et al.* 2020), it could be the most likely candidate for the salt tolerance in JP247291. However, it did not seem to explain the suppression of sodium allocation to the leaf in JP247291 (Fig. 1) as *POCO1* encoded a pentatricopeptide repeat (PRR) motif containing protein, which is one of the RNA-binding proteins. Thus, we considered there should be genes downstream of *POCO1*, which might be directly involved in regulation of SOS pathway or potassium transport.

Thus, we looked through the list of DEGs (*V. nakashimae* genome as a reference) that were significantly up-regulated or down-regulated throughout the three RNA-seq experiments (Supplemental Tables 11, 12). In the leaf, there were 1 up-regulated and 3 down-regulated genes, but none of them were directly related to salt tolerance. In the root, there were 41 up-regulated and 22 down-regulated genes. Of the up-regulated genes, we found *Vigna.01G00023500.01*, encoding STELAR K^+^ OUTWARD RECTIFIER (SKOR), which would be involved in ABA-induced potassium transport from root to the shoot (Demidchik 2014). We also found *Vigna.10G0002800.01*, encoding CBL-INTERACTING PROTEIN KINASE (CIPK), which is potentially involved in SOS1 activation (Yin *et al.* 2019).

## Discussion

In this study, we identified *POCO1* as a candidate gene for salt tolerance in a coastal species *V. nakashimae* by comparative transcriptome analysis with the assistance of forward genetic analysis and whole genome sequencing. Although JP254478 retains 3ʹ-end of the *POCO1* locus, it has lost transcriptional activity as the deletion has removed the promoter sequence (Fig. 5A, 5C). *POCO1* was first identified from an early-flowering mutant of Arabidopsis and was found to be a positive regulator of ABA signaling (Emami and Kempken 2019). As the *poco1* mutant exhibits reduced tolerance to drought (Emami and Kempken 2019) and transcription of genes related to ABA signaling, water stress and oxidative stress (Emami *et al.* 2020), we consider the reduced transcription of the *POCO1* gene is responsible for the relatively lower salt tolerance in JP254478. This argument is further supported by the positive correlation between transcriptional activity and salt tolerance in other species/accessions (Fig. 5B). In azuki bean, the *poco1* has lost its coding ability as well as its transcriptional activity (Fig. 5B, 5C). This is why we failed in detecting *POCO1* in the first transcriptome analysis and could be partially why azuki bean is not tolerant to salt stress.

Given *POCO1* is not a direct player of sodium transport, we consider it might be involved in positive regulation of SOS pathway or Na/K homeostasis. The mechanism of salt tolerance is suppressing Na allocation to the leaf by, to some extent, accumulating lots of K in the leaf and maintaining its Na/K ratio low (Noda *et al.* 2022). Interestingly, *Vigna.01G00023500.01*, which encodes SKOR protein, is actively transcribed in JP247291 even in the control condition (Supplemental Table 12). As SKOR is positively regulated by ABA and is involved in loading K from the root stele to xylem sap (Demidchik 2014), it may directly contribute to the higher ability of Na/K homeostasis in the leaf of *V. nakashimae*. In addition, *Vigna.10G0002800.01*, which encodes an ortholog of Arabidopsis CIPK8, is also actively transcribed in JP247291 (Supplemental Table 12). As CIPK8 has potential to activate SOS1 as SOS2 does (Yin *et al.* 2019), it could contribute to Na exclusion from the leaf cells. However, we need further studies to test whether these genes are in the regulatory network of *POCO1* and are truly responsible for the mechanism of salt tolerance.

It might also be intriguing to study if there is a merit in losing *POCO1*. Although JP254478 is not as tolerant to salt stress as JP247291 is, it grows much faster than JP247291 under the control condition (Supplemental Fig. 1). In addition, the growth rate of *V. angularis*, including wild azuki bean, is even bigger than *V. nakashimae* whereas that of *V. riukiuensis* is one of the smallest across the whole genus (Iseki *et al.* 2016). We would be willing to test whether loss of *POCO1* gene could reduce sensitivity to ABA and increase growth rate in the near future.

However, as we have not done any functional analysis of *POCO1* in the background of Vigna plants, it is still possible that other genes, which are not transcriptionally different between JP254478 and JP247291, are responsible for salt tolerance. One of such genes is *Vigna.08G0393700.01* that encodes another CIPK family protein and is potentially involved in Na/K homeostasis (Tang *et al.* 2020). However, none of those functionally potential candidates do not respond to salt stress in either of the accessions (Supplemental Tables 5–10). As such, we consider it is less likely that they are responsible for the difference of salt tolerance between the two accessions.

Besides identification of the candidate gene, there are lessons we should learn from this study. First, one should not ignore position effect even when plants are grown in a growth chamber (Measures *et al.* 1973). Even though the condition can be strictly controlled, the airflow from the duct does not evenly blow the plants. Plants placed at the edge of the hydroponic culture pools are more directly blown and are likely to suffer from higher transpiration-dependent sodium uptake (Fig. 2E, Supplemental Figs. 3, 4). In contrast, those in the middle are in the safest condition and it could be why some of the F_3_ lines with ‘SS’ genotype showed even lower damage score than JP247291 (Fig. 1E). However, if such position effect is minimized, one is able to maximize the proportion of genetic effect on the phenotypic variation in the mapping population, making the QTL analysis more reliable (Fig. 2D).

Second, one should be aware of the risk of relying on the genome sequences of a related species as a reference. As we encountered in our transcriptome analysis, the gene of interest could be missing or translocated even in the genomes of the related species where overall synteny is highly conserved (Figs. 3, 5C). The best solution to avoid such problems is to sequence, assemble and annotate one’s own material. This is now highly affordable, given the cost of long-read sequencing has dramatically reduced recently (De Coster *et al.* 2021). Having chromosome-level genome assemblies greatly facilitates the study, as we have easily identified structural variations between JP254478 and JP247291 (Figs. 4B, 4C, 5C).

Third, one should repeatedly perform RNA-seq experiments, including plant cultivation and RNA extraction, at least twice. As shown in Fig. 3, most of the DEGs detected in one experiment were not repeatedly detected as DEGs in other experiments. This could be due to biases in experimental skills or environmental effects in each experiment. We would not say that the non-overlapping DEGs across the experiments were false-positives, but each RNA-seq experiment might have detected many genes that could respond to conditional changes other than salt stress. In addition, although qPCR is often used to confirm the results of RNA-seq, doing RNA-seq and qPCR on the same RNA samples is almost nonsense because it is nothing but a validation of technology and not a validation of expression profile. If one is to test the reproducibility of RNA-seq results and cannot afford another sequencing, one has to do qPCR on RNA samples that were independently prepared from a replicated experiment, and not on those prepared for sequencing. However, as shown in Figs. 3 and 5, replicating RNA-seq experiments might often save one from looking through thousands of DEGs and greatly facilitates identification of the genes of interest, because truly-differentially expressed genes are always detected. The cost of short-reads also keeps declining every year (De Coster *et al.* 2021) and thus it is now practical to perform duplicated or triplicated RNA-seq experiments.

To conclude, although we had encountered problems and failures, the recently-lowered cost of sequencing technologies finally enabled us to find a candidate gene of salt tolerance from an accession of *V. nakashimae*. Given wild species are almost inexhaustible resources of tolerance to any kind of stress, it will be promising to explore them for more valuable genes. We hope this study could be a good example for such future studies.

## Author Contribution Statement

KN, YT, EOT and YM conceived the study and designed experiments. EOT developed the mapping population. HO and YT grew F_2_ plants and harvested F_3_ seeds. HO,YN and JF performed tracer experiments. HO, YT, AJN and KN performed genotyping and QTL analysis. MI, MT, TS, CM and KN performed RNA-seq experiments. MI and KN analyzed transcriptome data. KN performed whole genome sequencing, assembly and gene annotation. MI and KN wrote the paper.

## Supplementary Material

Supplemental Figures

Supplemental Tables

## Figures and Tables

**Table 1.** List of plant materials

Species	Name	Origin	Purpose	Salt tolerance
*V. angularis*	cv. Shumari	Hokkaido, Japan	RNA-seq	Sensitive
*V. nakashimae*	JP254478	Bueyo, South Korea	QTL, RNA-seq	Relatively sensitive
	JP247291	Ukushima Island, Japan	QTL, RNA-seq	Tolerant
	JP254455	Ukushima Island, Japan	RNA-seq	Tolerant
*V. riukiuensis*	JP254537	Ishigaki Island, Japan	RNA-seq	Tolerant

## References

[B1] Broman, K.W., D.M. Gatti, P. Simecek, N.A. Furlotte, P. Prins, Ś. Sen, B.S. Yandell and G.A. Churchill (2019) R/qtl2: Software for mapping quantitative trait loci with high-dimensional data and multiparent populations. Genetics 211: 495–502. 30591514 10.1534/genetics.118.301595 PMC6366910

[B2] Brůna, T., K.J. Hoff, A. Lomsadze, M. Stanke and M. Borodovsky (2021) BRAKER2: Automatic eukaryotic genome annotation with GeneMark-EP+ and AUGUSTUS supported by a protein database. NAR Genom Bioinform 3: lqaa108. 33575650 10.1093/nargab/lqaa108 PMC7787252

[B3] Chen, Y., A.T.L. Lun and G.K. Smyth (2016) From reads to genes to pathways: Differential expression analysis of RNA-Seq experiments using Rsubread and the edgeR quasi-likelihood pipeline. F1000Res 5: 1438. 27508061 10.12688/f1000research.8987.1 PMC4934518

[B4] Chen, Y., F. Nie, S.-Q. Xie, Y.-F. Zheng, Q. Dai, T. Bray, Y.-X. Wang, J.-F. Xing, Z.-J. Huang, D.-P. Wang et al. (2021) Efficient assembly of nanopore reads via highly accurate and intact error correction. Nat Commun 12: 60. 33397900 10.1038/s41467-020-20236-7 PMC7782737

[B5] De Coster, W., M.H. Weissensteiner and F.J. Sedlazeck (2021) Towards population-scale long-read sequencing. Nat Rev Genet 22: 572–587. 34050336 10.1038/s41576-021-00367-3 PMC8161719

[B6] Demidchik, V. (2014) Mechanisms and physiological roles of K^+^ efflux from root cells. J Plant Physiol 171: 696–707. 24685330 10.1016/j.jplph.2014.01.015

[B7] Emami, H. and F. Kempken (2019) PRECOCIOUS1 (POCO1), a mitochondrial pentatricopeptide repeat protein affects flowering time in *Arabidopsis thaliana*. Plant J 100: 265–278. 31219634 10.1111/tpj.14441

[B8] Emami, H., A. Kumar and F. Kempken (2020) Transcriptomic analysis of *poco1*, a mitochondrial pentatricopeptide repeat protein mutant in *Arabidopsis thaliana*. BMC Plant Biol 20: 209. 32397956 10.1186/s12870-020-02418-z PMC7216612

[B9] Hart, A.J., S. Ginzburg, M. Xu, C.R. Fisher, N. Rahmatpour, J.B. Mitton, R. Paul and J.L. Wegrzyn (2020) EnTAP: Bringing faster and smarter functional annotation to non-model eukaryotic transcriptomes. Mol Ecol Resour 20: 591–604. 31628884 10.1111/1755-0998.13106

[B10] Hassani, A., A. Azapagic and N. Shokri (2020) Predicting long-term dynamics of soil salinity and sodicity on a global scale. Proc Natl Acad Sci USA 117: 33017–33027. 33318212 10.1073/pnas.2013771117 PMC7776813

[B11] Iseki, K., Y. Takahashi, C. Muto, K. Naito and N. Tomooka (2016) Diversity and evolution of salt tolerance in the genus *Vigna*. PLoS One 11: e0164711. 27736995 10.1371/journal.pone.0164711 PMC5063378

[B12] Isemura, T., A. Kaga, S. Tabata, P. Somta, P. Srinives, T. Shimizu, U. Jo, D.A. Vaughan and N. Tomooka (2012) Construction of a genetic linkage map and genetic analysis of domestication related traits in mungbean (*Vigna radiata*). PLoS One 7: e41304. 22876284 10.1371/journal.pone.0041304 PMC3410902

[B13] Kaga, A., T. Isemura, N. Tomooka and D.A. Vaughan (2008) The genetics of domestication of the azuki bean (*Vigna angularis*). Genetics 178: 1013–1036. 18245368 10.1534/genetics.107.078451 PMC2248364

[B14] Keilwagen, J., F. Hartung, M. Paulini, S.O. Twardziok and J. Grau (2018) Combining RNA-seq data and homology-based gene prediction for plants, animals and fungi. BMC Bioinformatics 19: 189. 29843602 10.1186/s12859-018-2203-5 PMC5975413

[B15] McCarthy, D.J., Y. Chen and G.K. Smyth (2012) Differential expression analysis of multifactor RNA-Seq experiments with respect to biological variation. Nucleic Acids Res 40: 4288–4297. 22287627 10.1093/nar/gks042 PMC3378882

[B16] McCouch, S., G.J. Baute, J. Bradeen, P. Bramel, P.K. Bretting, E. Buckler, J.M. Burke, D. Charest, S. Cloutier, G. Cole et al. (2013) Feeding the future. Nature 499: 23–24. 23823779 10.1038/499023a

[B17] Measures, M., P. Weinberger and H. Baer (1973) Variability of plant growth within controlled environment chambers as related to temperature and light distribution. Can J Plant Sci 53: 215–220.

[B18] Naito, K. (2023) How to sequence and assemble plant genomes. Methods Mol Biol 2632: 57–77. 36781721 10.1007/978-1-0716-2996-3_5

[B19] Noda, Y., R. Sugita, A. Hirose, N. Kawachi, K. Tanoi, J. Furukawa and K. Naito (2022) Diversity of Na^+^ allocation in salt-tolerant species of the genus *Vigna*. Breed Sci 72: 326–331. 36699821 10.1270/jsbbs.22012 PMC9868329

[B20] Panta, S., T. Flowers, P. Lane, R. Doyle, G. Haros and S. Shabala (2014) Halophyte agriculture: Success stories. Environ Exp Bot 107: 71–83.

[B21] Patro, R., G. Duggal, M.I. Love, R.A. Irizarry and C. Kingsford (2017) Salmon provides fast and bias-aware quantification of transcript expression. Nat Methods 14: 417–419. 28263959 10.1038/nmeth.4197 PMC5600148

[B22] Pertea, G. and M. Pertea (2020) GFF Utilities: GffRead and GffCompare. F1000Res 9. 10.12688/f1000research.23297.1 PMC7222033 32489650

[B23] Pokhrel, Y., F. Felfelani, Y. Satoh, J. Boulange, P. Burek, A. Gädeke, D. Gerten, S.N. Gosling, M. Grillakis, L. Gudmundsson et al. (2021) Global terrestrial water storage and drought severity under climate change. Nat Climate Change 11: 226–233.

[B24] Roach, M.J., S.A. Schmidt and A.R. Borneman (2018) Purge Haplotigs: Allelic contig reassignment for third-gen diploid genome assemblies. BMC Bioinformatics 19: 460. 30497373 10.1186/s12859-018-2485-7 PMC6267036

[B25] Rochette, N.C., A.G. Rivera-Colón and J.M. Catchen (2019) Stacks 2: Analytical methods for paired-end sequencing improve RADseq-based population genomics. Mol Ecol 28: 4737–4754. 31550391 10.1111/mec.15253

[B26] Sakaguchi, S., T. Sugino, Y. Tsumura, M. Ito, M.D. Crisp, D.M.J.S. Bowman, A.J. Nagano, M.N. Honjo, M. Yasugi, H. Kudoh et al. (2015) High-throughput linkage mapping of Australian white cypress pine (*Callitris glaucophylla*) and map transferability to related species. Tree Genet Genomes 11: 121.

[B27] Sakai, H., K. Naito, E. Ogiso-Tanaka, Y. Takahashi, K. Iseki, C. Muto, K. Satou, K. Teruya, A. Shiroma, M. Shimoji et al. (2015a) The power of single molecule real-time sequencing technology in the *de novo* assembly of a eukaryotic genome. Sci Rep 5: 16780. 26616024 10.1038/srep16780 PMC4663752

[B28] Sakai, H., K. Naito, Y. Takahashi, T. Sato, T. Yamamoto, I. Muto, T. Itoh and N. Tomooka (2015b) The *Vigna* Genome Server, ‘*Vig*GS’: A genomic knowledge base of the genus *Vigna* based on high-quality, annotated genome sequence of the azuki bean, *Vigna angularis* (Willd.) Ohwi & Ohashi. Plant Cell Physiol 57: e2. 26644460 10.1093/pcp/pcv189

[B29] Tang, R.-J., C. Wang, K. Li and S. Luan (2020) The CBL-CIPK calcium signaling network: Unified paradigm from 20 years of discoveries. Trends Plant Sci 25: 604–617. 32407699 10.1016/j.tplants.2020.01.009

[B30] Vasimuddin, M., S. Misra, H. Li and S. Aluru (2019) Efficient Architecture-Aware Acceleration of BWA-MEM for Multicore Systems2019 IEEE International Parallel and Distributed Processing Symposium (IPDPS).

[B31] Wada, Y., L.P.H. van Beek, C.M. van Kempen, J.W.T.M. Reckman, S. Vasak and M.F.P. Bierkens (2010) Global depletion of groundwater resources. Geophys Res Lett 37: L20402.

[B32] Wang, L., S. Kikuchi, C. Muto, K. Naito, T. Isemura, M. Ishimoto, X. Cheng, A. Kaga and N. Tomooka (2015) Reciprocal translocation identified in *Vigna angularis* dominates the wild population in East Japan. J Plant Res 128: 653–663. 25796202 10.1007/s10265-015-0720-0

[B33] Yao, F., B. Livneh, B. Rajagopalan, J. Wang, J.-F. Crétaux, Y. Wada and M. Berge-Nguyen (2023) Satellites reveal widespread decline in global lake water storage. Science 380: 743–749. 37200445 10.1126/science.abo2812

[B34] Yin, X., Y. Xia, Q. Xie, Y. Cao, Z. Wang, G. Hao, J. Song, Y. Zhou and X. Jiang (2019) The protein kinase complex CBL10–CIPK8–SOS1 functions in Arabidopsis to regulate salt tolerance. J Exp Bot 71: 1801–1814. 10.1093/jxb/erz549 PMC7242078 31858132

[B35] Yoshida, Y., R. Marubodee, E. Ogiso-Tanaka, K. Iseki, T. Isemura, Y. Takahashi, C. Muto, K. Naito, A. Kaga, K. Okuno et al. (2016) Salt tolerance in wild relatives of adzuki bean, *Vigna angularis* (Willd.) Ohwi et Ohashi. Genet Resour Crop Evol 63: 627–637.

