# Supplementary material for: Single candidate gene for salt tolerance of Vigna nakashimae (Ohwi) Ohwi & Ohashi identified by QTL mapping, whole genome sequencing and triplicated RNA-seq analyses
Source: Breed Sci. 2024 Mar 22;74(2):93–102. doi: 10.1270/jsbbs.23053 (PMC11442111; doi:10.1270/jsbbs.23053)
Supplement: Supplementary file 1 — Supplemental Figures [file 74_093_s1.pdf]

Ito et al./Breeding Science
doi: 10.1270/jsbbs.23053

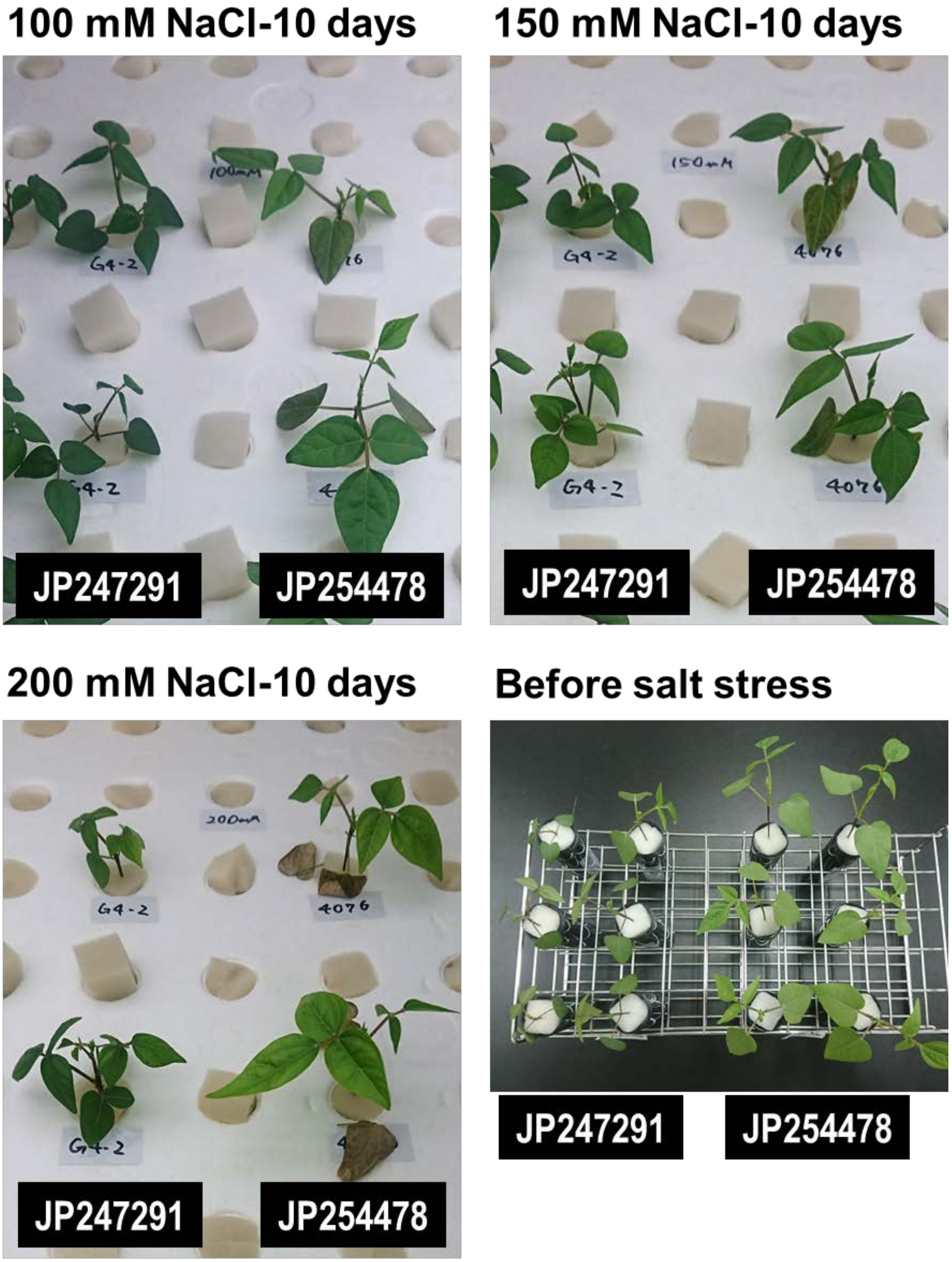

Supplemental Figure 1. Plants of parental accessions before and after 200 mM NaCl treatment.

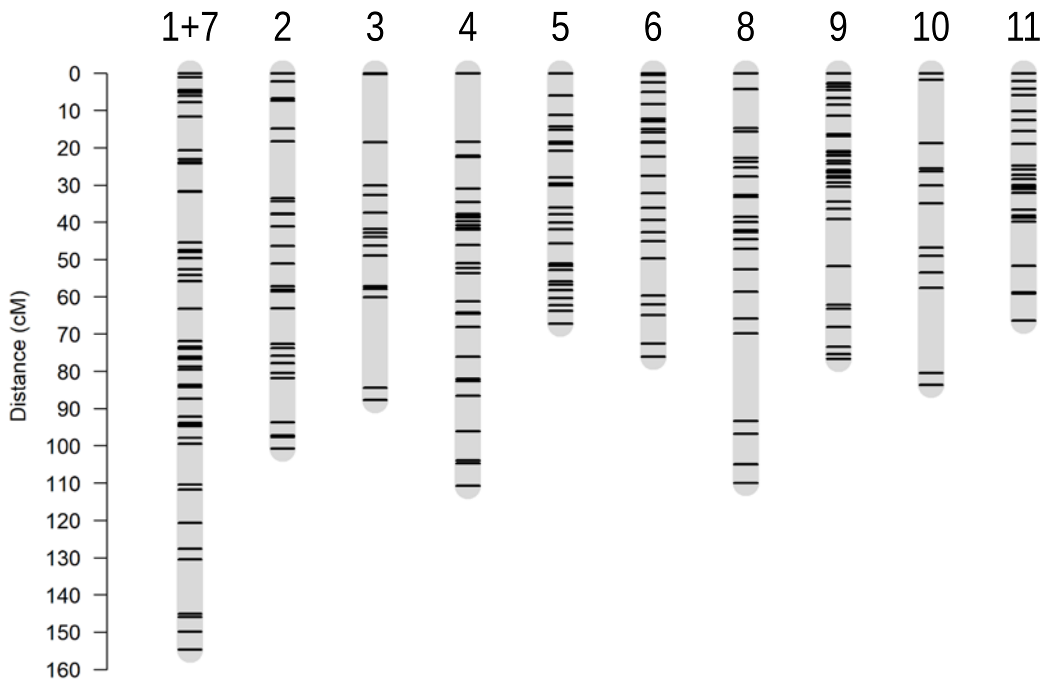

Supplemental Figure 2. Genetic linkage map of *Vigna nakashimae*. Horizontal lines indicate marker loci.

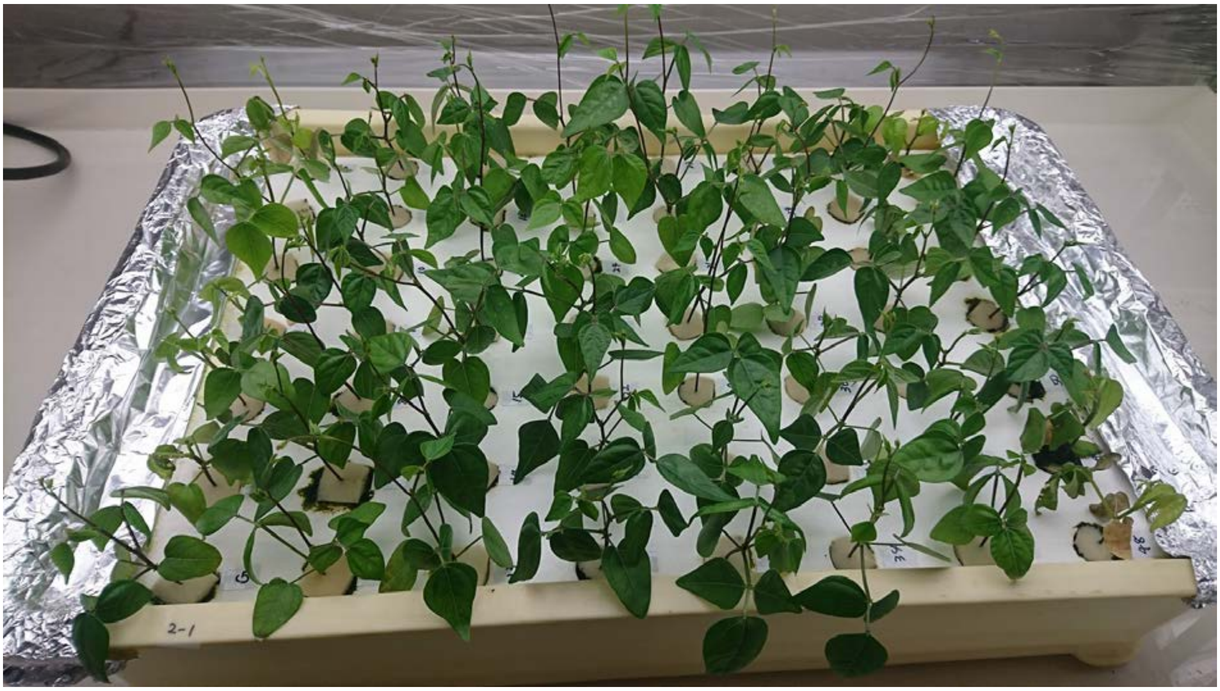

Supplemental Figure 3. F3 plants exhibiting position effect. The photo was taken 10 days after transfer to the condition of 200 mM NaCl. The damage seemed higher in the plants placed at the edge compared to those in the middle.

Wilt scores of the F3 plants at the edge of the culture pools

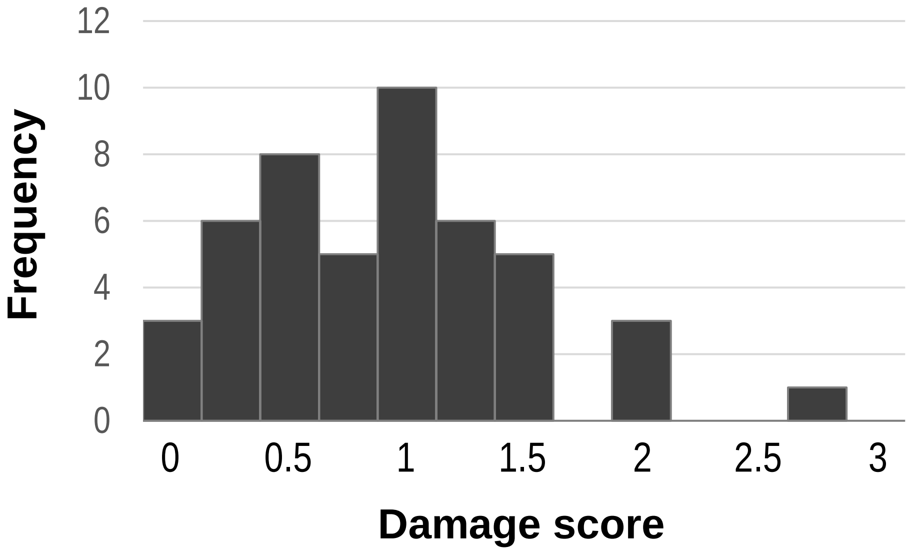

Wilt scores of the F3 plants inside the culture pools

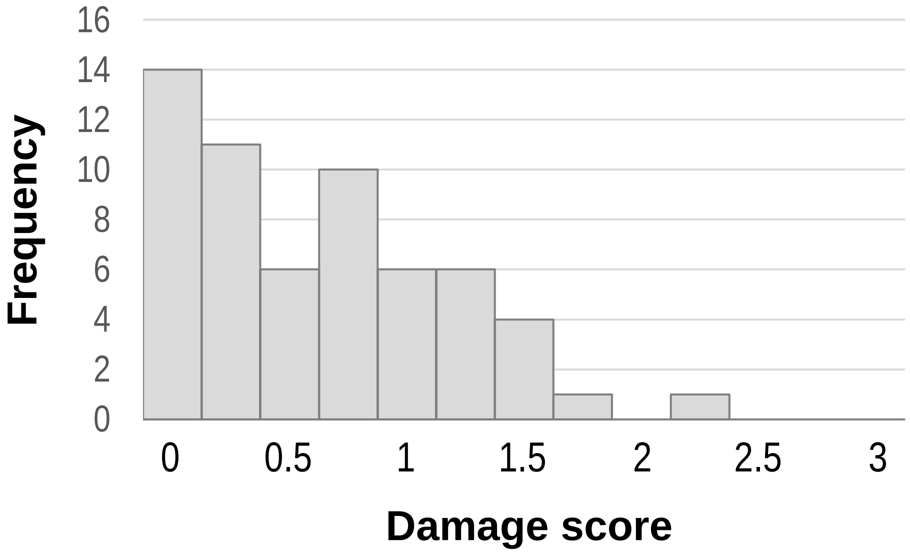

Values of Mann-Whitney U test

U = 980.0, var = 24699.9, Z = 2.59, p = 0.009695

Supplemental Figure 4. Distribution of damage scores in F3 plants placed at the edge of or inside the hydroponic culture pool.
